# Hyperspectral Imaging for the Evaluation of Microcirculatory Tissue Oxygenation and Perfusion Quality in Haemorrhagic Shock: A Porcine Study

**DOI:** 10.3390/biomedicines9121829

**Published:** 2021-12-03

**Authors:** Maximilian Dietrich, Berkin Özdemir, Daniel Gruneberg, Clara Petersen, Alexander Studier-Fischer, Maik von der Forst, Felix C. F. Schmitt, Mascha O. Fiedler, Felix Nickel, Beat Peter Müller-Stich, Thorsten Brenner, Markus A. Weigand, Florian Uhle, Karsten Schmidt

**Affiliations:** 1 Department of Anesthesiology, Heidelberg University Hospital, 69120 Heidelberg, Germany; daniel.gruneberg@med.uni-heidelberg.de (D.G.); clara.petersen@stud.uni-heidelberg.de (C.P.); Maik.Forst@med.uni-heidelberg.de (M.v.d.F.); Felix.Schmitt@med.uni-heidelberg.de (F.C.F.S.); Mascha.Fiedler@med.uni-heidelberg.de (M.O.F.); Markus.Weigand@med.uni-heidelberg.de (M.A.W.); Florian.Uhle@med.uni-heidelberg.de (F.U.); 2 Department of General, Visceral and Transplantation Surgery, Heidelberg University Hospital, 69120 Heidelberg, Germany; kamiloe32@gmail.com (B.Ö.); Alexander.Studier-Fischer@med.uni-heidelberg.de (A.S.-F.); Felix.Nickel@med.uni-heidelberg.de (F.N.); BeatPeter.Mueller@med.uni-heidelberg.de (B.P.M.-S.); 3 Department of Anesthesiology and Intensive Care Medicine, University Hospital Essen, University Duisburg-Essen, 45147 Essen, Germany; Thorsten.Brenner@uk-essen.de (T.B.); Karsten.Schmidt@uk-essen.de (K.S.)

**Keywords:** hyperspectral imaging, shock, haemorrhage, microcirculation, resuscitation, haemodynamic therapy, monitoring

## Abstract

Background: The ultimate goal of haemodynamic therapy is to improve microcirculatory tissue and organ perfusion. Hyperspectral imaging (HSI) has the potential to enable noninvasive microcirculatory monitoring at bedside. Methods: HSI (Tivita^®^ Tissue System) measurements of tissue oxygenation, haemoglobin, and water content in the skin (ear) and kidney were evaluated in a double-hit porcine model of major abdominal surgery and haemorrhagic shock. Animals of the control group (n = 7) did not receive any resuscitation regime. The interventional groups were treated exclusively with either crystalloid (n = 8) or continuous norepinephrine infusion (n = 7). Results: Haemorrhagic shock led to a drop in tissue oxygenation parameters in all groups. These correlated with established indirect markers of tissue oxygenation. Fluid therapy restored tissue oxygenation parameters. Skin and kidney measurements correlated well. High dose norepinephrine therapy deteriorated tissue oxygenation. Tissue water content increased both in the skin and the kidney in response to fluid therapy. Conclusions: HSI detected dynamic changes in tissue oxygenation and perfusion quality during shock and was able to indicate resuscitation effectivity. The observed correlation between HSI skin and kidney measurements may offer an estimation of organ oxygenation impairment from skin monitoring. HSI microcirculatory monitoring could open up new opportunities for the guidance of haemodynamic management.

## 1. Introduction

Haemodynamic therapy aims to maintain tissue perfusion and oxygen delivery to protect organ function during phases of circulatory compromise [1]. A prerequisite for effective resuscitation therapy is haemodynamic coherence, in which attaining macrohaemodynamic target parameters translates into the restoration of sufficient microcirculatory perfusion and tissue oxygenation [2]. Bedside monitoring technology that recognises tissue perfusion and oxygenation deficits, as well as detrimental fluid and vasopressor effects, could improve outcomes in high-risk surgical and critically ill patients. However, monitoring methods that provide direct microcirculatory feedback are not widely incorporated into perioperative clinical practice [3].

Hyperspectral imaging (HSI) is an evolving noninvasive imaging technology that provides an evaluation of intrinsic biochemical tissue characteristics based on tissue-light interactions [4]. HSI technologies are increasingly being investigated in surgical research for real-time intraoperative organ perfusion-based resection planning and optimization of anastomosis quality [5,6,7]. Data on HSI technologies for microcirculatory monitoring of haemodynamic therapy in critically ill or surgical patients are limited. The TIVITA^®^ Tissue camera system (Diaspective Vision GmbH, Am Salzhaff, Germany) provides noninvasive HSI for qualitative and quantitative bedside microcirculatory assessment. The key feature of this HSI camera system is a set of four parameters that allow a differentiated and spatially resolved investigation of tissue oxygenation, perfusion quality, haemoglobin distribution, and tissue water content [4,8]. Studies in wound care and surgery demonstrated the HSI system’s ability to detect clinically relevant short- and long-term changes in tissue oxygenation and perfusion quality [9,10]. First experiences point to the feasibility of this HSI system detecting clinically relevant microcirculatory skin alterations in critically ill patients with sepsis [11].

In a double-hit porcine model of major abdominal surgery and haemorrhagic shock, we aimed to evaluate whether HSI can detect pathological skin and kidney tissue oxygenation and perfusion deficits, the response to fluid and vasopressor therapy, and detrimental resuscitation side effects. Furthermore, correlations between skin and kidney HSI findings, as well as correlations to macrohaemodynamic variables and global markers of tissue oxygenation, should be evaluated.

## 2. Methods

This study implemented on 27 pigs (Sus scrofa) was approved by the appropriate governmental body (Regierungspräsidium Karlsruhe, file reference G-261/19) and conducted in accordance with the European law on the protection of animals used for scientific purposes (EU-Directive 2010/63). The study reporting adheres to the ARRIVE guidelines 2.0 [12].

### 2.1. Sample Size Calculation, Randomisation, and Blinding

Serum lactate after one hour of therapy following shock induction was used as the effect variable for sample size calculation. A decrease of 25%, compared with the control group, was considered biologically and clinically relevant. For calculations (G* Power V3.1.9 software, HHU Düsseldorf, Germany), a standard deviation of 20%, a significance level of α = 0.05, and a power of 1-β = 0.8 were applied, resulting in an estimated sample size of 8 animals per group. There was no preferred sex. All animals were assigned to the treatment groups in advance. The investigator who selected the animals for the experiment was not aware of the group assignment. Experimenters were not blinded to the group assignment.

### 2.2. Animal Preparation and Anaesthesia

All pigs were kept inside in the Interfacultary Biomedical Facility of the University of Heidelberg at a constant temperature and a controlled circadian rhythm. They fasted the day of surgery. Water was accessible until anaesthesia induction. The pigs were premedicated with 1 mg/kg midazolam (Midazolam-hameln^®^ 5 mg/mL by hameln pharma plus Gmbh^®^, Hameln, Germany) and 10 mg/kg ketamine (Ketamin 10%^®^ by Heinrich Fromme^®^, Warburg, Germany). Pre-medication was administered by intramuscular injection to the neck. In unconscious pigs, an auricular vein catheter was placed. For endotracheal intubation (Willy Rüsch GmbH, Kernen, Germany), the animal was placed in a prone position, and 2 mg/kg of propofol was administered. Throughout the experiment, anaesthesia was maintained with a combination of intravenous 0.5–1 mg/kg/h midazolam, 10–20 mg/kg/h ketamine, and inhalational Sevoflurane^®^ (exsp.vol% 1.5–2.5). Depth of anaesthesia was confirmed regularly by the lack of spontaneous movements and absence of reaction and cardiovascular signs to surgery or painful stimulation between the front hooves.

### 2.3. Ventilatory Settings

Volume-controlled ventilation (Respirator: Primus^®^ Dräger Medical AG, Lübeck, Germany) with a tidal volume of 8 mL/kg, a positive-end-expiratory pressure of 5 mbar, an inspiration-to-expiration ratio (I:E ratio) of 1:2, and a fixed FiO_2_ of 0.5 was performed. The respiratory rate was adjusted to reach an end-tidal CO_2_ of 40 ± 5 mmHg. Peripheral oxygen saturation was monitored with a saturation probe fixed to the tail.

### 2.4. Haemodynamic Monitoring

The right external jugular vein and the right femoral artery were surgically prepared. A central venous catheter (ARROW^®^ 3-Lumen Central Venous Catheter, Wayne, PA, USA) and an arterial PiCCO catheter (PULSION Medical Systems SE, Feldkirchen, Germany) were implemented to allow continuous haemodynamic monitoring including heart rate, blood pressure, stroke volume variability, cardiac output measurement (thermodilution measurements consisted of three boluses of 20 mL NaCl 0.9% (B. Braun SE, Melsungen, Germany) with a temperature of 4 °C, and blood gas analysis. Body temperature was sustained with electrical heat blankets and monitored with an oesophageal temperature probe.

## 3. Experimental Protocol

### 3.1. Surgical Procedure

Following midline laparotomy, the right kidney was exposed for HSI measurements. To simulate a major abdominal surgical procedure, the pigs were then splenectomised, and a mobilisation procedure of the stomach with dissection of attached ligaments was performed.

### 3.2. Shock Induction

After completing the surgical procedure, haemorrhagic shock was induced by drawing blood from the central venous catheter. The amount of blood removed was adjusted to achieve a target mean arterial pressure (MAP) of 40 ± 5 mmHg for 60 min.

### 3.3. Haemodynamic Management and Intervention

**Figure 1:**
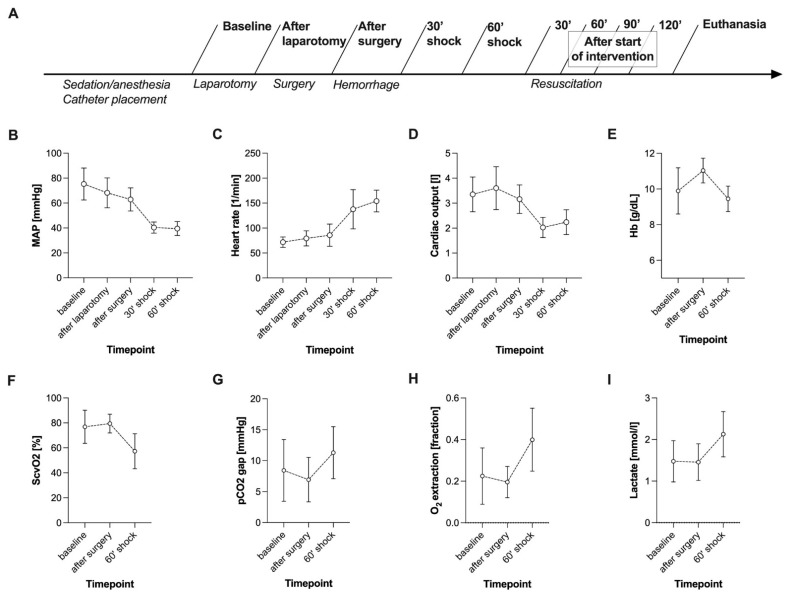
Experimental timeline (**A**) and course of macrohaemodynamics (**B**–**E**) and global markers of tissue oxygenation (**F**–**I**) during surgery and haemorrhagic shock induction. Data of all groups (n = 22) are presented together. Haemorrhagic shock was induced after the surgical procedure was completed with a target MAP of 40 ± 5 mmHg. Data are shown as mean with standard deviation; MAP: mean arterial pressure; HB: haemoglobin level; ScvO2: central venous oxygen saturation.

All pigs received a basic fluid rate of 10 mL/kg/h crystalloid infusion (Sterofundin ISO^®^ by B. Braun^®^, Melsungen, Germany). The control group (CG) did not receive a specific haemodynamic treatment regime following shock induction. The fluid-treated group (FG) received a continuous crystalloid infusion (Sterofundin^®^, B. Braun SE, Melsungen, Germany). The norepinephrine-treated group (NG) received a continuous infusion of noradrenaline (Arterenol^®^, Sanofi-Aventis Deutschland GmbH, Höchst, Germany). In both FG and NG, a MAP of 65 mmHg was targeted for the first treatment period of 60 min and a MAP of 90 mmHg for the second treatment period of 60 min (Figure 1A). Fluid and norepinephrine therapy were titrated by the attending experimenter to reach the aforementioned MAP targets.

### 3.4. Euthanasia

The anaesthetised pigs were euthanised with potassium chloride. Death was confirmed with ECG and etCO_2_.

### 3.5. HSI Measurements

Hyperspectral imaging (HSI) was performed with the Tivita^®^ Tissue System (Diaspective Vision GmbH, Am Salzhaff, Germany). The technical specifications of the system have been described in previous publications [4,8,9]. The system uses 100 wavelengths for the calculation of the following HSI parameters:

- Tissue oxygenation (StO_2_, wavelength range: 500–650 and 700–815 nm): haemoglobin oxygen saturation in the capillary system of superficial tissue (penetration depth up to 1 mm) indicated in percent (0–100%);
- NIR perfusion index (NIR, wavelength range: 655–735 and 825–925 nm): haemoglobin oxygen saturation in the capillary system of deeper tissue layers (penetration depth up to 4–6 mm) indicated in predefined arbitrary units (0–100);
- Tissue haemoglobin index (THI, wavelength range: 530–590 and 785–825 nm): distribution of deoxygenated and oxygenated haemoglobin in the measured tissue (penetration depth up to 1–3 mm) indicated in predefined arbitrary units (0–100);
- Tissue water index (TWI, wavelength range: 880–900 and 955–980 nm): relative water content of the tissue (penetration depth up to 1–3 mm) indicated in predefined arbitrary units (0–100).

HSI measurements were conducted on the inside of one ear (skin measurement site) and the right kidney. The camera-specific software (TIVITA^®TM^ Suite) was used to define a circular region of interest (ROI) in each HSI image to obtain objective numerical values. One unit of the circular ROI equals the radius in pixels. The largest possible circular area of homogenous tissue uninterrupted by any lesions, plicae, or light reflections was selected. ROI size at the ear and the kidney was 30 and 20 arbitrary units (Figure S1), respectively. To standardise picture acquisition conditions, the system’s integrated sensor was used to warn of disturbing stray light. The target device with two overlapping light points ensured the maintenance of a 50 cm distance between the investigated tissue and the camera. The experimental setup is illustrated in Figure S2.

### 3.6. Data Collection Time Points

Data of HSI measurements and vital parameter documentation were collected at nine time points (Figure 1A). Data collection at baseline, after completion of the surgical procedure, after 60 min of haemorrhage, after 60 min of intervention, and after 120 min of intervention included arterial and central venous blood gas analysis.

### 3.7. Statistical Methods

Data were collected with the aid of an electronic database system (Microsoft Excel^®^, Microsoft Deutschland GmbH, Unterschleißheim, Germany). GraphPad Prism (Version VIII, GraphPad Software, La Jolla, CA, USA, Graph Pad) was used for statistical analyses. Descriptive statistics were performed for the complete dataset. Mean and standard deviation are presented. Spearman’s correlation analyses were used. Metric data over courses of time and between intervention groups were evaluated by analyses of covariance with a mixed effect model. For the comparison of paired samples, the Wilcoxon test was used.

## 4. Results

In total, 27 pigs (weight 35.6 kg ± 2.6 kg) were assessed in the study, and data from 22 animals (CG n = 7, FG n = 8, NG = 7) were analysed. Three pigs died during the observation period, and two were excluded from the analysis due to severely impaired haemodynamics at baseline.

### 4.1. Haemorrhagic Shock Induced Global and HSI-Measured Tissue Oxygenation Deficiency

**Figure 2:**
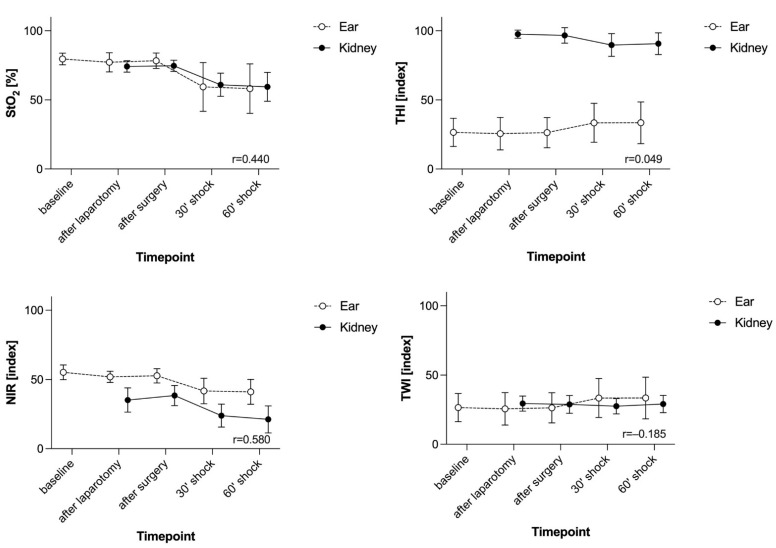
The course of parameters derived from HSI during surgery and haemorrhagic shock induction at the skin and the kidney. Data of all groups (n = 22) are presented together. StO_2_ is given in %. THI, NIR, and TWI are index values in an arbitrary unit. Haemorrhagic shock was induced after the surgical procedure was completed with a target MAP of 40 ± 5 mmHg. Data are shown as mean with standard deviation. The correlation coefficient r is shown in the bottom right-hand corner of the diagram. Spearman’s correlation analysis was used; HSI: hyperspectral imaging, StO_2_: tissue oxygenation, THI: tissue haemoglobin index, NIR: near-infrared perfusion index, TWI: tissue water index.

**Figure 3:**
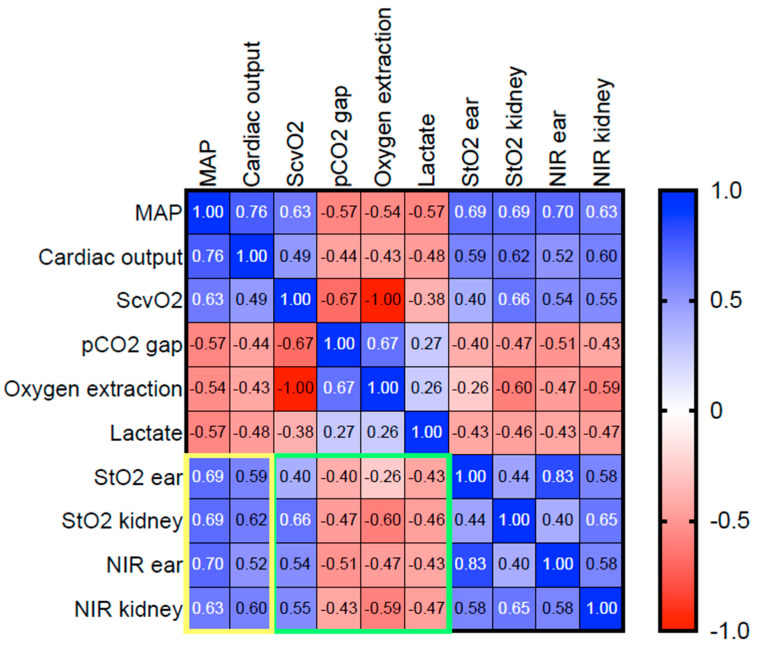
Heat map of the correlation analyses of macro- and microcirculatory parameters during surgery and haemorrhagic shock induction. Spearman’s correlation analysis was used. Positive and negative correlations are displayed blue and red, respectively. A stronger shade indicates a stronger correlation. Data of all groups (n = 22) were analysed together. The yellow and the green squares highlight the correlation of HSI-derived tissue oxygenation markers with macro- and microcirculatory parameters, respectively. MAP: mean arterial pressure, CO: cardiac output, ScvO_2_: central venous oxygen saturation, HSI: hyperspectral imaging, StO_2_: tissue oxygenation, THI: tissue haemoglobin index, NIR: near-infrared perfusion index, TWI: tissue water index.

During the major abdominal surgical procedure, all animals showed stable macrohaemodynamic parameters (Figure 1B–D). Surgical trauma did not affect the HSI parameters at the skin or the kidney (Figure 2). MAP targeted induction of haemorrhagic shock (40 ± 5 mmHg) required a mean blood withdrawal of 967 mL ± 169 mL and led to reduced cardiac output and compensatory high heart rate (Figure 1B–D). Simultaneously, the pCO_2_ gap, oxygen extraction, and lactate increased, while central venous oxygen saturation (ScvO_2_) decreased (Figure 1F–I). In response to haemorrhagic shock induction, HSI tissue oxygenation parameters StO_2_ and NIR of the skin and the kidney dropped significantly (surgery complete vs. 60’shock *p* < 0.0001, Figure 2). During surgery and shock induction, HSI skin and kidney oxygenation correlated moderately with each other (Figure 2), as well as with macrohaemodynamic indices (Figure 3, yellow box) and global markers of tissue oxygenation deficiency (Figure 3, green box).

### 4.2. MAP Targets Were Not Consistently Achieved during Fluid and Vasopressor Therapy

**Figure 4:**
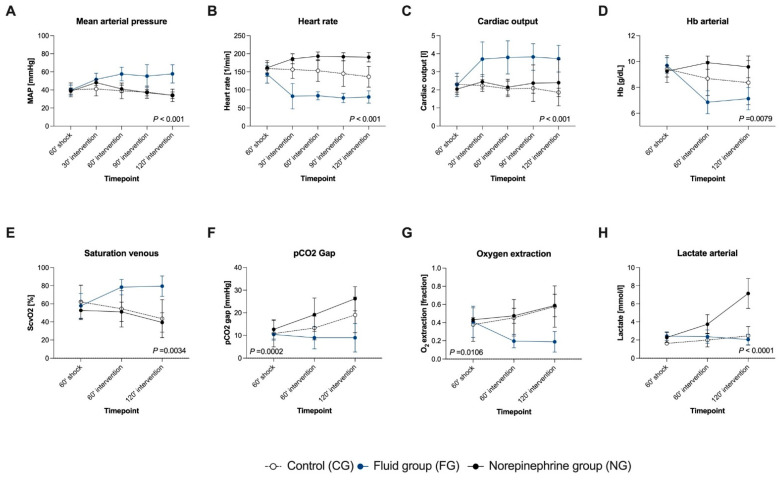
The course of macrohaemodynamics (**A**–**D**) and global markers of tissue oxygenation (**E**–**H**) during the interventional resuscitation phase. The target MAP in both interventional groups was 65 mmHg in the first and 90 mmHg in the second hour of intervention. Data are shown as mean with standard deviation. The groups were analysed separately (CG: n = 7, FG: n = 8, NG: n = 7). The *p*-value of the analysis of covariance with a mixed effect model is shown in the diagram; MAP: mean arterial pressure, HB: haemoglobin level, ScvO2: central venous oxygen saturation.

In both intervention groups (FG and NG), the MAP targets of 65 mmHg in the first and 90 mmHg in the second hour of intervention were not achieved (Figure 4A).

### 4.3. Fluid Resuscitation Restored Global and HSI-Measured Tissue Oxygenation

**Figure 5:**
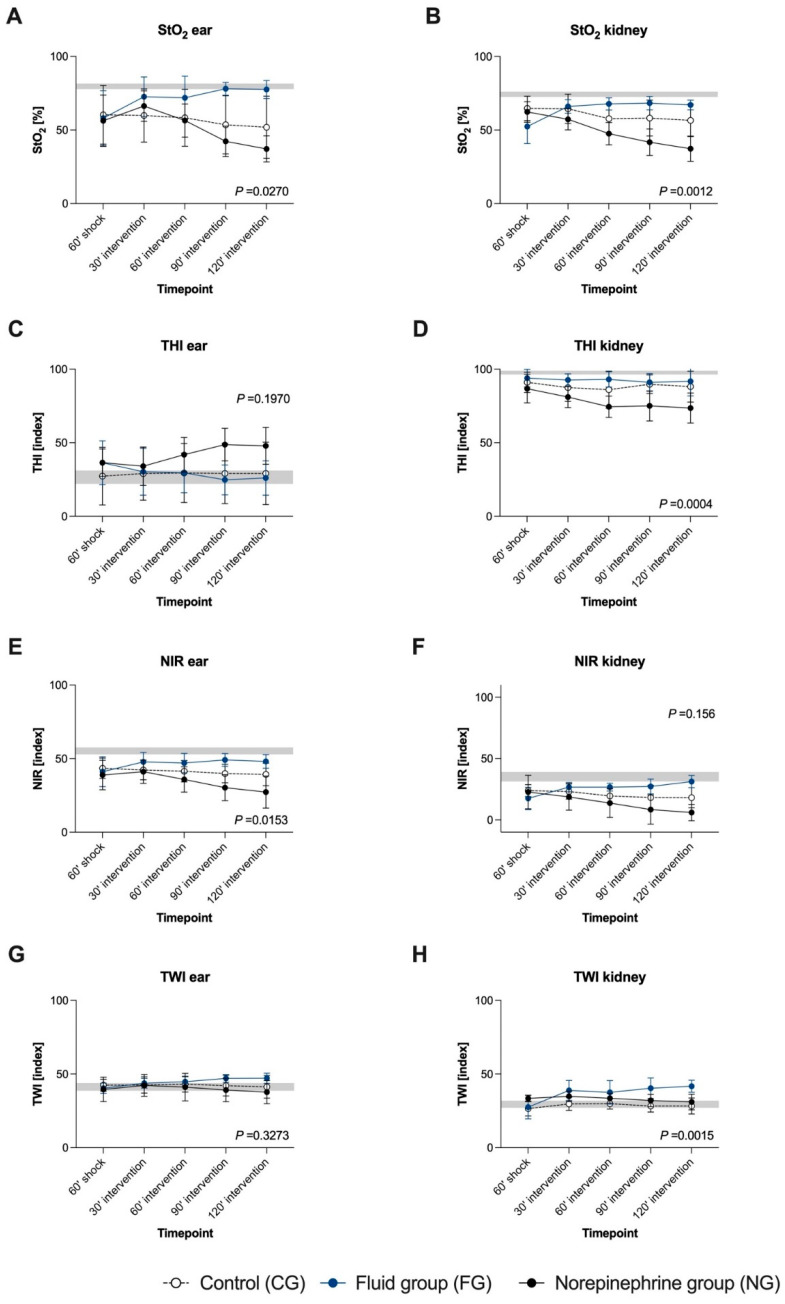
The course of parameters derived from HSI during the interventional resuscitation phase at the skin and the kidney. The target MAP in both interventional groups was 65 mmHg in the first and 90 mmHg in the second hour of intervention. StO_2_ (**A**,**B**) is given in %. THI (**C**,**D**), NIR (**E**,**F**), and TWI (**G**,**H**) are index values in an arbitrary unit. Data are shown as mean with standard deviation. The groups were analysed separately (CG: n = 7, FG: n = 8, NG: n = 7). The grey background area indicates the initial value range (min–max). The *p*-value of the analysis of covariance with a mixed effect model is shown in the diagram; HSI: hyperspectral imaging, StO_2_: tissue oxygenation, THI: tissue haemoglobin index, NIR: near-infrared perfusion index, TWI: tissue water index.

In FG, fluid therapy significantly increased cardiac output and MAP, compared with CG and NG (Figure 4A–C). Intervention in FG led to a decrease in oxygen extraction and an increase in ScvO2 (Figure 4E,G). HSI tissue oxygenation parameters StO_2_ (Figure 5A,B) and NIR (Figure 5E,F) rose significantly in the FG and remained on a higher level than in CG and NG, both at the skin and the kidney. THI was not affected by fluid administration (Figure 5C,D).

### 4.4. Fluid Administration Increased HSI-Measured Tissue Water Content

TWI of the skin and the kidney increased (Figure 5G,H) and correlated strongly with the cumulative amount of administered fluid in the FG (mean cumulative given fluid: 30’: 3239 ± 666 mL; 60’: 4524 ± 999 mL; 90’: 5745 ± 968 mL; 120’: 6774 ± 1099 mL) (TWI skin r = 0.61, *p* < 0.0001; TWI kidney r = 0.64, *p* < 0.0001).

### 4.5. Norepinephrine Administration Aggravated Global and HSI Tissue Oxygenation Deficiency Dose-Dependently

Increasing doses of norepinephrine (30’: 0.73 ± 0.17 µg/kg/min; 60’: 1.03 ± 0.08 µg/kg/min; 90’: 1.36 ± 0.22 µg/kg/min; 120’: 1.92 ± 0.44 µg/kg/min) induced a persisting rise in heart rate, whereas MAP fell again after a transient increase at 30’ after intervention start (Figure 4A). Noradrenaline administration did not induce major changes in cardiac output (Figure 4C). The pCO_2_ gap and lactate increased dose-dependently in the NG, whereas no differences in ScvO_2_ and oxygen extraction between NG and CG were observed (Figure 4E–H).

StO_2_ of the skin and the kidney, as well as skin NIR (Figure 5E) were significantly lower in the NG, compared with both other groups (Figure 5A,B). THI at the kidney decreased continuously in the NG (Figure 5D). In contrast, there was a trend towards an ascent of THI at the skin (Figure 5C). TWI did not differ between NG and CG (Figure 5G,H).

### 4.6. Macrohaemodynamic Variables and Global Markers of Tissue Oxygenation Correlated with HSI Tissue Oxygenation Parameters during Resuscitation

**Figure 6:**
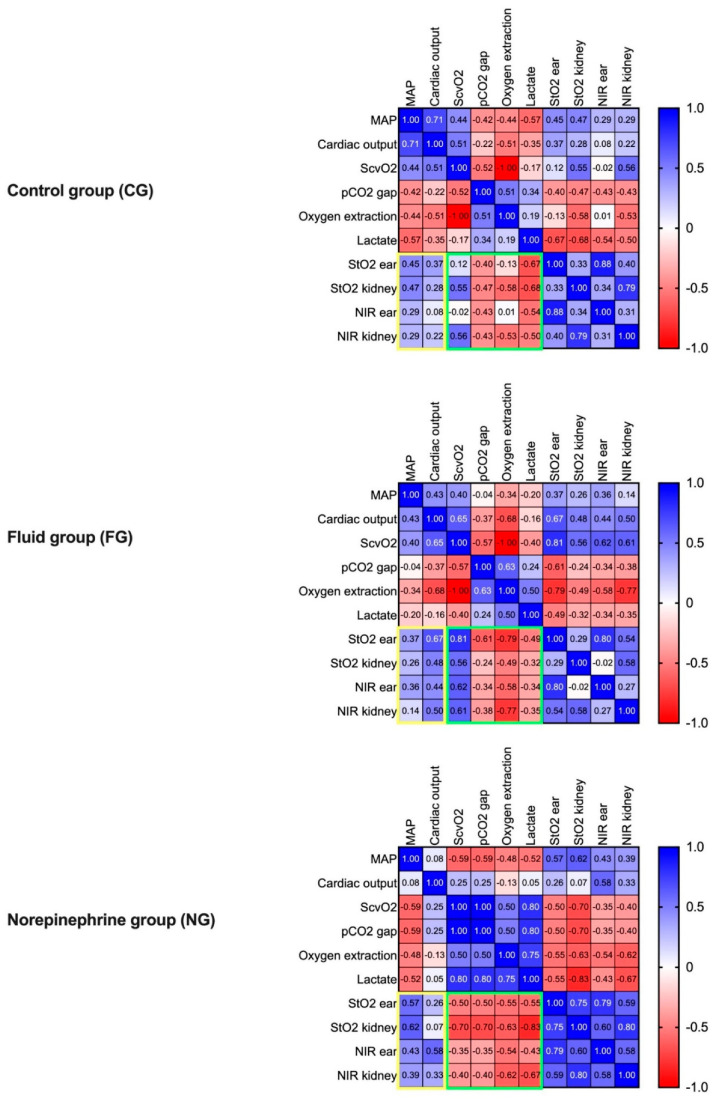
Heat maps of the correlation analyses of macro- and microcirculatory parameters individually for each group during the interventional resuscitation phase. The groups were analysed separately (CG n = 7, FG: n = 8, NG: n = 7). Spearman’s correlation analysis was used. Positive and negative correlations are displayed in blue and red, respectively. A stronger shade indicates a stronger correlation. In addition, the correlation coefficient is displayed for each analysis. The yellow and the green squares highlight the correlation of HSI-derived tissue oxygenation markers with macro- and microcirculatory parameters, respectively; MAP: mean arterial pressure; CO: cardiac output, ScvO2: central venous oxygen saturation, HSI: hyperspectral imaging, StO_2_: tissue oxygenation, THI: tissue haemoglobin index, NIR: near-infrared perfusion index, TWI: tissue water index.

In the FG, HSI tissue oxygenation parameters StO_2_ and NIR correlated strongly with cardiac output and weakly to moderately with MAP. Contrariwise, a moderate to strong correlation with MAP was observed in the NG (Figure 6, yellow box). In all groups, there was a moderate, negative correlation of StO_2_ and NPI with the pCO_2_ gap and lactate, both at the skin and the kidney (Figure 6, green box). In the CG, ScvO_2_ and oxygen extraction correlated with StO_2_ and NPI at the kidney but not at the skin. In the NG, there was a negative correlation between HSI tissue oxygenation markers assessed at the skin and kidney and ScvO_2_, in sharp contrast to FG, where fluid resuscitation induced a strong positive correlation of ScvO_2_ with StO_2_ and NIR. In both interventional groups, there was a strong negative correlation of StO_2_ and NIR with oxygen extraction. During intervention phases, there was a weak correlation of StO_2_ and NIR between the two measurement sites of ear and kidney in CG and FG. In the NG, a strong correlation between HSI oxygenation parameters of the ear and kidney was observed.

## 5. Discussion

One obstacle for the perioperative introduction of tissue-perfusion-guided haemodynamic therapy is the limited monitoring capability that provides feedback about microcirculatory function. HSI is a noninvasive bedside technology that offers information about major determinants of microcirculatory function such as oxygenation, perfusion quality, tissue haemoglobin, as well as tissue water content [4,8,9]. We used HSI for the evaluation of skin and kidneys in a double-hit porcine model of major abdominal surgery and haemorrhagic shock, followed by MAP-guided resuscitation therapy. We report that HSI detects skin and kidney tissue oxygenation and perfusion deficits that correlate with pathological changes of global markers of tissue oxygenation during haemorrhagic shock. Furthermore, we demonstrate that HSI-tracked the microcirculatory response to fluid and vasopressor resuscitation. Changes in HSI parameters and global markers of tissue oxygenation and their respective correlations indicated resuscitation effectivity. In addition, changes in HSI parameters indicated detrimental effects of fluid and vasopressor therapy.

Tissue hypoxia is a major pathophysiological outcome determinant in both high-risk surgical and critically ill patients. In this study, haemorrhage led to a marked decrease in HSI tissue oxygenation parameters StO_2_ and NIR at the skin and the kidney. During shock and resuscitation, StO_2_ and NIR correlated with changes in macrohaemodynamics and global markers of oxygenation. These findings indicate that HSI is capable of detecting clinically relevant tissue oxygenation deficiency. Haemorrhagic shock is a subcategory of hypovolemic shock. During shock, the animals in our study demonstrated low cardiac output and increased haemoconcentration, in line with intravascular volume depletion. Fluid therapy resulted in haemodilution, restored cardiac output, and improved tissue oxygenation. Based on our results, we propose that the observed microcirculatory effect is essentially due to the reversal of the intravascular fluid deficit. Therefore, we suggest that our findings could be applied to hypovolemic shock in general. The transferability of our findings to other shock forms, such as distributive or cardiogenic shocks, needs further investigation.

Tissue-perfusion-guided therapy provides the opportunity to prevent organ dysfunction by identifying diminished or persisting oxygen delivery to tissues. An ongoing matter of debate is the appropriate measurement site for microcirculatory monitoring because microcirculatory alterations in one organ do not necessarily reflect the situation in other organs. Here, we report a correlation of HSI oxygenation parameters of the skin and the kidney during shock induction and resuscitation. Cancio et al. investigated changes in skin oxygenation in a porcine model of haemorrhagic shock with a different HSI system at the inner hindlimb as a measurement site. In line with our results, a reduction in skin oxygenation during shock was observed. However, organ tissue oxygenation was not assessed [13]. Langeland et al. showed a uniform decrease in muscle, gut, and kidney blood flow during haemorrhagic shock, whereas skin microcirculation was not evaluated [14]. Hutchings et al. reported an association of microvascular hypoperfusion measured with a sidestream dark field technique in traumatic haemorrhagic shock patients with multiorgan dysfunction [15]. Previous studies in septic patients showed an association of skin oxygenation derived from HSI and organ failure [16]. Our data indicate that HSI could be used as a clinically relevant monitoring tool during circulatory compromise and haemodynamic therapy by providing tissue oxygenation feedback. We propose that intraoperative HSI skin monitoring alone or combined with organ measurements in the surgical field could be used as a surrogate for appropriate tissue oxygen supply. Further studies should evaluate the relationship between HSI-derived tissue oxygenation and haemoglobin content and direct capillary flow measurements to investigate the pathophysiological background of the changes in microvascular flow in shock.

We chose this porcine shock model to evaluate the capability of HSI to detect microcirculatory resuscitation effectivity and detrimental side effects of fluid and vasopressor therapy. While aware of the limitations of MAP-targeted resuscitation therapy, we used two MAP levels as endpoints during two treatment intervals in FG and NG to identify HSI patterns that could indicate under- or over-resuscitation [17].

Fluid resuscitation resulted in haemodynamic stabilisation and reduction in oxygen supply deficiency. This was paralleled by the return of decreased HSI tissue oxygenation parameters StO_2_ and NIR to approximately preshock levels. StO_2_ and NIR correlated stronger with cardiac output than with MAP during fluid therapy. In a previous study, Langeland et al. showed a coherent behaviour of cardiac output, kidney, muscle, and gut microcirculatory blood flow, and tissue metabolic response in a porcine haemorrhagic shock model [14]. In our study, the changes in cardiac output within the first 30 minutes of fluid treatment indicate fluid responsiveness of the hypovolemic pigs. We suppose that the concomitant StO_2_ and NIR changes reflect microcirculatory flow responsiveness.

The administration of norepinephrine without an additional fluid resuscitation in the NG did not result in attaining MAP targets despite steady dose escalations. Moreover, HSI tissue oxygenation parameters StO_2_ and NIR, pCO2 gap, and lactate indicated a dose-dependent aggravation of shock-induced tissue oxygenation deficiency. Conceptually, pigs in the NG were fluid under- and vasopressor over-resuscitated. Norepinephrine can lead to excessive vasoconstriction which may result in an impairment of tissue perfusion [18]. We observed a specific HSI pattern in the NG indicative of detrimental vasopressor-induced tissue hypoperfusion. This finding could provide clinically relevant information, especially in critically ill patients requiring increased vasopressor therapy. Vasopressor effects are dependent on the pathophysiology of shock and the vascular bed under evaluation [19,20]. HSI could be capable of differentiating complex haemodynamic patterns by combined evaluation of parameters for oxygenation and perfusion quality (StO_2_/NIR) and distribution of oxy-and deoxygenated haemoglobin (THI). Our group observed that septic nonsurvivors had significantly elevated THI on admission to the intensive care unit—these patients showed increased vasopressor dependency [11]. Previous surgical HSI data propose that a rising THI is indicative of venous congestion in flaps [21]. We proposed that an elevated THI could be interpreted as a red blood cell pooling in the skin microcirculation indicative of disturbed perfusion or stagnant flow situation [11].

In this study, we observed that skin and kidney THI showed no relevant changes during haemorrhagic shock induction. In the NG, THI revealed opposite behaviour following norepinephrine administration: skin THI increased and kidney THI decreased, whereas StO_2_ and NIR showed a parallel decrease at the skin and kidney in the NG. One possible interpretation could be that the increased tissue haemoglobin on the skin reflects reduced capillary efflux with consecutive capillary congestion. In combination with low tissue oxygenation parameters, THI could be useful in assessing perfusion quality and potentially warn physicians of the detrimental effects of excessive catecholamine administration. The correlation of oxygenation parameters between the skin and the kidney measurement site was stronger under norepinephrine than fluid treatment. The reason for that could lie in compensatory mechanisms of centralisation, in which central organs are preferentially supplied with blood during shock. Administration of norepinephrine could affect these mechanisms by altering the perfusion distribution between peripheral and organ tissues, thereby worsening perfusion quality in both tissues equally, resulting in a better correlation and high haemoglobin content of the skin [20].

Fluid overload and concomitant tissue oedema formation are dreaded detrimental effects of fluid therapy [22,23]. There is no established bedside method to measure changes in tissue water content in perioperative and critical care medicine. Tissue hypoxia in oedema is fuelled by increased oxygen diffusion distances due to the accumulation of tissue water. HSI technology allows real-time visualisation and evaluation of tissue water content based on the specific light absorption characteristics of water [4,24]. We previously observed in septic patients a significantly increased palm TWI, compared with healthy controls [11]. TWI was not affected during shock induction in this study. We observed that fluid administration progressively increased TWI in both the skin and the kidney. Furthermore, the cumulative amount of fluid correlated with TWI in the FG. Following 60 minutes of therapy, no further improvements of HSI tissue oxygenation parameters, macrohaemodynamics, and global tissue oxygenation markers could be observed in response to fluids, but TWI continued to increase. Therefore, we propose that TWI could indicate progressive tissue oedema formation and should be further investigated as a possible stop sign for fluid administration.

## 6. Conclusions

HSI detected dynamic changes in tissue oxygenation and perfusion quality during shock and was able to indicate resuscitation effectivity. Further, HSI measurements of skin oxygenation reflected the situation in the kidney well and thus support the hypothesis to monitor skin microcirculatory alterations during circulatory compromise to infer organ oxygenation adequacy. We propose that distinctive HSI parameter patterns could enable tissue-perfusion-guided therapy targeting StO_2_ and NIR. THI and TWI could potentially be useful to warn of detrimental effects of vasopressor and fluid therapy, respectively. The combined evaluation of tissue oxygenation, haemoglobin, and water content could open up new opportunities for the guidance of haemodynamic management, which has to be substantiated in future interventional trials.

## Data Availability

Data are made available on reasonable request through the principal investigators.

## Supplementary Materials

The following are available online at https://www.mdpi.com/article/10.3390/biomedicines9121829/s1, Figure S1: Exemplary RGB and colour-coded HSI images for the control, norepinephrine, and fluid group of the tissue oxygenation parameter StO_2_ with regions of interest (ROI) of the ear and kidney at baseline, after 60 min of shock, and after 120 min of intervention, Figure S2: Schematic representation of the experimental setup.
