# Supplementary material for: Hyperspectral Imaging for the Evaluation of Microcirculatory Tissue Oxygenation and Perfusion Quality in Haemorrhagic Shock: A Porcine Study
Source: Biomedicines. 2021 Dec 3;9(12):1829. doi: 10.3390/biomedicines9121829 (PMC8698916; doi:10.3390/biomedicines9121829)
Supplement: Supplementary file 1 [file biomedicines-09-01829-s001.zip › biomedicines-1473851-supplementary.pdf]

biomedicines

MDPI

Supplementary Materials

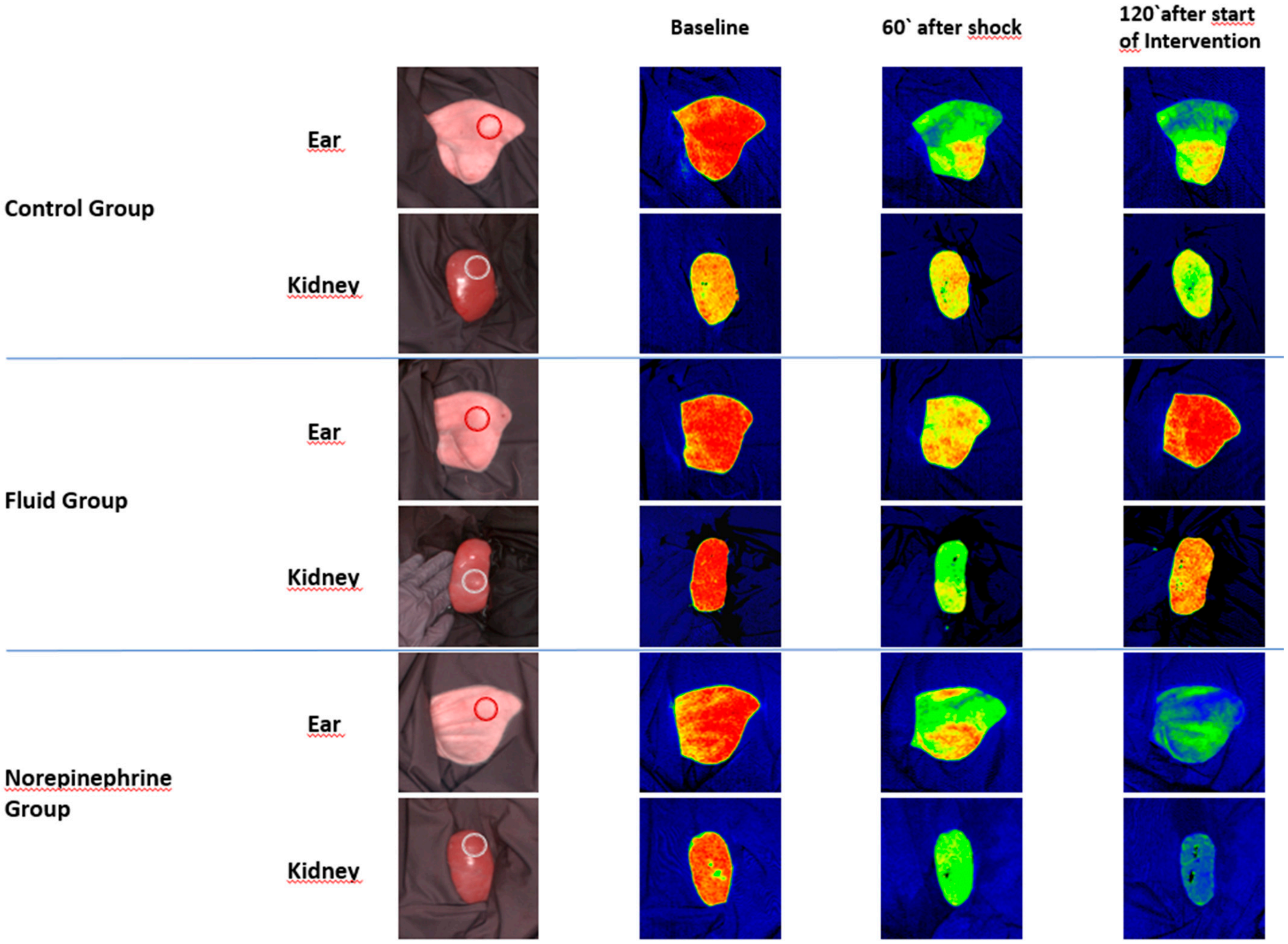

Supplement Figure S1: Exemplary RGB and colour-coded HSI images for the Control, Norepinephrine and Fluid Group of the tissue oxygenation parameter StO2 with regions of interest (ROI) of the ear and kidney at baseline, after 60 minutes of shock and after 120 minutes of intervention; Tissue oxygenation (StO2) is displayed color-coded. Red/yellow areas indicate high values (50-100%), green/blue (0-50%) areas indicate low values. The circles drawn are the region of interest from which the numerical values are obtained. HSI: Hyperspectral imaging, RGB: Red-green-blue.

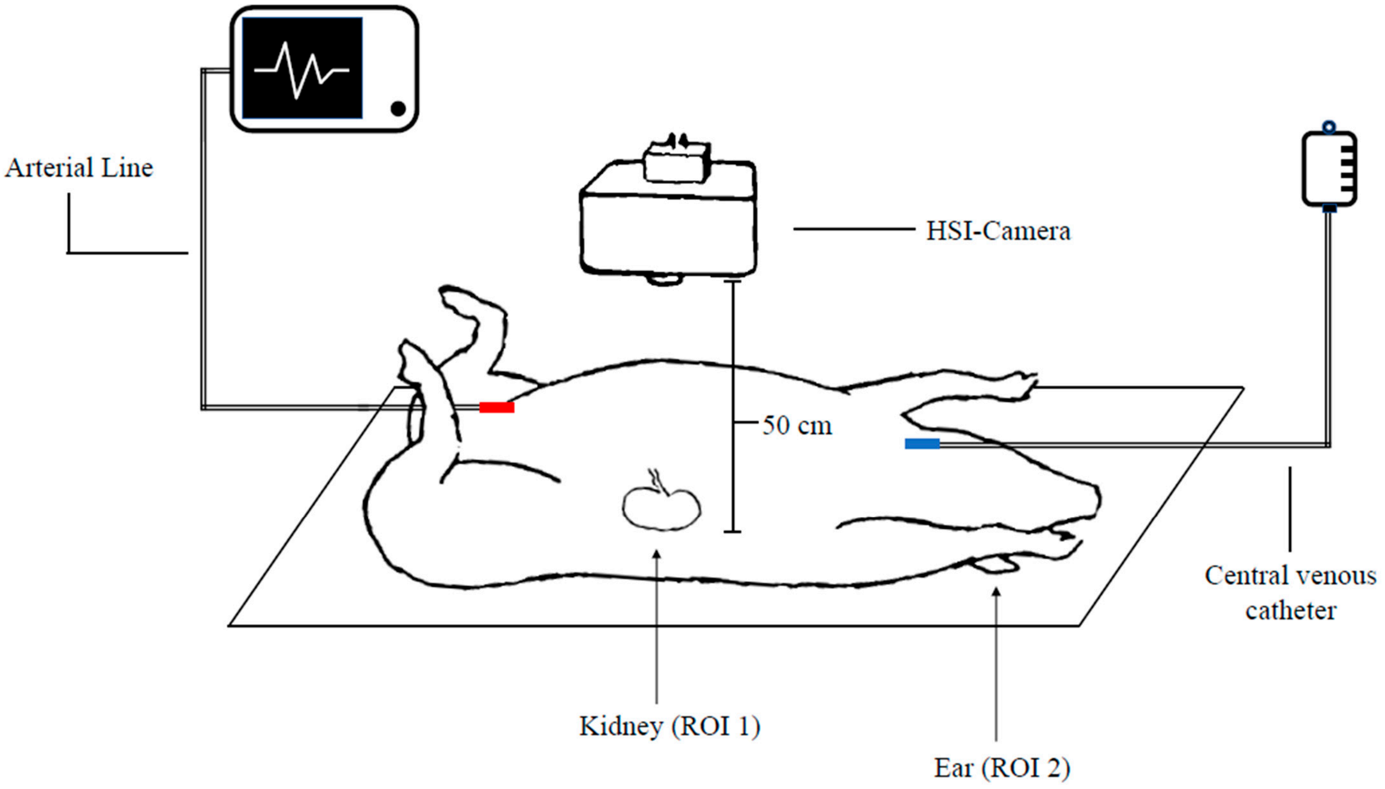

Supplement Figure S2: Schematic representation of the experimental setup.
